# Why are there different types of synesthete?

**DOI:** 10.3389/fpsyg.2013.00558

**Published:** 2013-09-02

**Authors:** Julia Simner

**Affiliations:** Department of Psychology, School of Philosophy, Psychology and Language Sciences, University of EdinburghEdinburgh, UK

**Keywords:** individual differences, visual imagery, synaesthesia, synesthesia, projector, associator

For people with synesthesia, sensations in two modalities are experienced when only one is stimulated (e.g., auditory stimuli might trigger colors and sounds). Synesthetes are manifestly different to the general population, but can also be different to each other. First, the condition is widely heterogeneous in that 60–150 different manifestations of synesthesia have been identified (e.g., auditory stimuli might trigger colors, shapes, flavors and so on; Cytowic and Eagleman, 2009). Second, synesthetes can differ on the *quality* of their synesthetic perceptions even within a given variant. Some experience their synesthetic percepts as being similar in quality to a real-world perceptions (e.g., synesthetic colors might be projected onto external objects and be difficult to dissociate from real-world colors) while other synesthetes experience less “veridical” percepts (see below). In this opinion piece I ask whether this particular difference—known as the “projector” vs. “associator” distinction—might fall out naturally from another, independent psychological quality.

The projector/associator distinction was first phrased (by Dixon et al., 2004) in terms of *grapheme-color synesthesia*, in which colors are triggered by letters and/or digits. Some grapheme-color synesthetes report that their colors are experienced outside their own body space, projected into the world, and these are termed “projector” synesthetes. Ward and colleagues further divide this category into two: “surface-projectors” experience color projected onto the written type-face (or more generally, onto the inducing stimulus whatever that might be), and “space-projectors” project color onto some other externalized near-space. In contrast to this external projection, other synesthetes (termed “associators”) can be thought of as “non-projectors” in that their colors exist within their internal mental space. Those associators who claim to see colors in their mind's eye have been termed “see-associators,” while those who simply claim to *know* the colors of graphemes without any associated impression of “seeing” at all have been termed “know-associators” (Ward et al., 2007). In other words there is a four-way divide between synesthetes experiencing colors in a way resembling real-world experiences (projected onto the stimulus or into near-space) and those experiencing them “internally” (in the mind's eye or as a type of propositional association). The claim put forward in this piece is a parsimonious one: that these differences might emerge from an otherwise unrelated individual difference, in the ability to form a visual mental image.

Visual imagery—the mental construction of a scene-like object—is known to vary across individuals (e.g., Marks, 1973). At the upper extreme end there are individuals with “eidetic” imagery who report strongly evoked mental images with an almost veridical quality. At the opposite end of the healthy spectrum are those who report being unable to form mental images at all. Taking these latter into consideration, and assuming a prevalence of synesthesia of at least 4% (Simner et al., 2006), we might assume—all other things being equal—that 4% of people with poor or no imagery abilities will have synesthesia. The proposal here is that these individuals are precisely those termed “know associators.” Equally, those with extreme imagery abilities might be those we recognise as projectors, because their high imagery allows their synesthetic associations to become “scene-like” to an extreme extent. Note that this view (if it were correct) would rely on the assumption that imagery and synesthesia are independent qualities and I now compare this view with current thinking in the literature.

Some researchers have proposed that synesthetes are in fact characterized, as a group, by superior mental imagery (e.g., Barnett and Newell, 2008; Price, 2009a,b), and some have gone so far as to ask whether some visual synesthesias may be *nothing more than* extreme visual imagery (Galton, 1880; Phillips, 1897; Price, 2009a). One well-phrased expression of a possible link between synesthesia and imagery is that normal cross-modal sensations (say, between numbers and space) may enter into consciousness only if the individual has heightened imagery (Eagleman, 2009; Price, 2009a), and this is precisely when he/she would become considered a “synesthete” (Simner, 2012). (The resultant synesthesia in this example would be *sequence-space synesthesia*—in which sequences such as numbers are consciously experienced in spatial arrays). In contrast with this view, others have suggested that synesthesia and imagery might be quasi-independent in that synesthetes might vary in their imagery ability, and hence some could have relatively weak imagery (see Grossenbacher and Lovelace, 2001, who put this view in passing).

Questions about synesthesia and imagery have also been tested empirically. Barnett and Newell (2008) showed that (*n* = 38) synesthetes with colored language (e.g., grapheme-color synesthesia) report significantly stronger vivid everyday mental images than controls, in a self-report questionnaire (*Vividness of Mental Imagery Questionnaire*, *VVIQ*, Marks, 1973). Meier and Rothen (under review) show similarly for (*n* = 24) grapheme-color synesthetes using the *Verbalizer-Visualizer Questionnaire* (VVQ; Kirby et al., 1988). And the same has been shown by Price (2009a) testing 12 sequence-space synesthetes, who outperformed controls in two questionnaires of visual imagery (*Subjective Use of Imagery Scale*, *SUIS*; Reisberg et al., 2003; and the visual [but not spatial] component of the *Object–Spatial Imagery Questionnaire*, *OSIQ*; Blajenkova et al., 2006). However, there has been some variation in the consistency of this self-reported superiority in imagery. Seron et al. (1992) failed to find higher than average visual imagery in 26 sequence-space synesthetes, using Paivio's *Individual Difference Questionnaire* (IDQ; Paivio, 1971). Furthermore, Spiller and Jansari (2008) failed to find a group difference in the VVIQ between six grapheme-color synesthetes and matched controls, although this may be due to low power.

In addition to poor consistency across synesthesia studies showing self-reported superiority in imagery, there has also been some variation in the extent to which this has translated into behavioral support. Despite no *self-reported* differences, grapheme-color synesthetes in Spiller and Jansari (2008) out-performed controls on a task that relied on mental imagery (imaging a grapheme within a divided circle and assessing which segment contained the majority of that image). Another study also found behavior suggesting synesthetes have superior imagery (e.g., sequence-space in 3D mental rotation; Simner et al., 2009). However, their group size was small (*n* = 5) and this effect was not replicated in a larger sample of nine synesthetes with a similar (sequence-space) variant (Rizza and Price, 2012). Nonetheless, Simner et al. tested somewhat unusual synesthetes with an unusually large array of synesthetic forms (e.g., for minutes, hours, days, months, numerals, letters, temperature etc.; mean = 7.0 forms; *SD* = 2.3). In contrast, Rizza and Price's subjects required only two forms to be selected for study. Superior performance in behavioral imagery tasks might therefore be tied to “superior” (more extreme) synesthesia. Nonetheless, a superiority in mental rotation for (*n* = 15) sequence-space synesthetes has now also been replicated by Brang et al. (2013; in 2D rotation), although across all three studies, we must conclude that the effect does not appear to be robust enough to survive repeated replication, especially where synesthetes might vary in their “strength.”

Finally, Spiller and Jansari found no correlation between their self-reported imagery scores and a behavioral visual imagery task, suggesting that self-report may not always reflect performance in lab-based imagery tests. Furthermore, Rizza and Price (2012) point out that mental rotation has not previously correlated with self-report in visual imagery, but rather *spatial* imagery, even though synesthetes do not report higher than average spatial skills (Rizza and Price, 2012; Meier and Rothen, under review). However, as Logie et al. (2011; p. 3072) “subjective reports [of mental imagery] do not always correlate with performance on mental imagery tasks … [perhaps because] people have poor insight into their mental operations when rating them.”

From these somewhat conflicting findings we can draw the following conclusions: although grapheme-color and sequence-space synesthetes have reported higher imagery in self-report visual imagery questionnaires, this self-report advantage has not been found consistently across studies of grapheme-color synesthetes (e.g., Barnett and Newell, 2008 vs. Spiller and Jansari, 2008 and Seron et al., 1992) and nor has it consistently translated into behavioral superiority for sequence-space synesthetes in mental rotation (e.g., Simner et al., 2009; Brang et al., 2013 vs. Rizza and Price, 2012). Furthermore, there is conflict both across and within studies between the predictions of self-report imagery questionnaires and behavioral tests such as imaging graphemes (for grapheme-color synesthetes; e.g., Spiller and Jansari, 2008) and mental rotation (for sequence-space synesthetes; e.g., Rizza and Price, 2012 vs. Simner et al., 2009 and Brang et al., 2013). Where conflicts arise across different cohorts of synesthetes with the same variant, we might conclude either that low power in some studies may be to blame, or subtle differences in methodologies, or indeed that meaningful individual differences in imagery might exist. In other words, if conflicts across studies represent genuine differences in imagery across synesthetes, then imagery and synesthesia may be either partially or fully independent.

If synesthesia and imagery are only partially independent, we might conclude that whatever causes synesthesia could perhaps sometimes have repercussions for high imagery, or vice versa, but that high imagery is not a necessary component of synesthesia. This would certainly be compatible with findings in functional magnetic resonance imaging (fMRI) that synesthetic colors are experienced with at least some differences in regions compared to the colors within the mental imagery of non-synesthetes (Rich et al., 2006). Partial independence could also explain why some synesthetes show no significant advantage in tests/questionnaires of imagery: if synesthesia and high imagery only *tend* to be linked, some cases would exist where the synesthete's imagery was low. However the tendency itself would also explain why there is an overall trend toward finding higher imagery across the body of literature as a whole (e.g., Barnett and Newell, 2008; Spiller and Jansari, 2008; Meier and Rothen, under review).

But let us now consider the possibility that these skills may be *fully* independent. If correct, this theory would smartly explain why some studies find no imagery advantages in their synesthetes. However, it would also need to explain why some synesthetes do score highly in imagery, and perhaps more often than we might expect by chance alone. Barnett and Newell (2008) and others have suggested that high imagery is a general characteristic of synesthesia but I would suggest it may simply be a characteristic of those synesthetes that self-report for testing. What then is different about these individuals? One consistent quality of all participants in the imagery studies reviewed here is that all were fully aware of their synesthesia (hence their self-referral and/or affirmations of synesthesia), but importantly, this need not be true of all synesthetes. It is reasonable to propose that an individual's awareness of synesthesia might be heightened if individual differences in imagery make synesthetic colors highly image-able for some synesthetes (but not others—who instead might simply “know” their associations at some propositional level). In other words, I suggest that individual and randomly dispersed variations in mental imagery within the synesthesia population would lead to some synesthetes having high imagery and therefore highly imaged synesthetic colors. This in turn could lead to enhanced conscious awareness, and consequently a greater likelihood of self-referral for scientific studies. This proposal is in opposition to the view that synesthetes overall are necessarily high imagers as a *sin qua non* and/or that synesthesia is nothing more than high mental imagery. (Indeed, this same self-referral argument might hold equally over other hypotheses, such as whether synesthetes have enhanced memory; e.g., Rothen et al., 2012).

In summary, I have proposed that empirical evidence is currently equivocal for synesthetes as high imagers. I have also suggested that trends toward high imagery in published studies might arise from a recruitment bias in which high-imaging synesthetes are more likely to self-refer. If high imagery is neither sufficient nor necessary for synesthesia, this would explain why some studies fail to show superior imagery, and would also nicely capture why some synesthetes describe their colors (or other sensations) as simply “known.” Put differently, for those without the ability to form a mental image, there would be no visual-like quality to their synesthetic sensations. This theory would also explain why other synesthetes do, in contrast, describe their colors as highly imaged, even eidetically so. My proposal sits alongside another theory, by Ward et al. (2007), that other differences within this same spectrum (e.g., surface vs. near-space projectors) could also fall out naturally from other *a priori* individual differences (this time from differences in spatial reference frames). In future studies it will be important to establish whether trends found in smaller populations are true even when larger numbers are assessed. It is also important to look at recruitment methods since self-referred samples might be expected to show stronger imagery than cohorts more randomly sampled. Finally, future studies might seek a standardized assessment of projector/associators, given that previous efforts to classify individuals have shown differences across labs (c.f., Dixon et al., 2004; Ward et al., 2007) and even over time (Edquist et al., 2006). A useful classification would also extend to all forms of synesthesia, including variants (e.g., sequence-space) in which the projector/associator distinction has been recognized thus far only in self-report.
